# Comparative Study on Mechanical Property and Fracture Behavior of Age-Hardened LM4 Monolithic Composites Reinforced with TiB_2_ and Si_3_N_4_

**DOI:** 10.3390/ma16113965

**Published:** 2023-05-25

**Authors:** Srinivas Doddapaneni, Sathyashankara Sharma, Gowrishankar Mandya Chennegowda, Manjunath Shettar, Ananda Hegde

**Affiliations:** Department of Mechanical and Industrial Engineering, Manipal Institute of Technology, Manipal Academy of Higher Education, Manipal 576104, India

**Keywords:** stir casting, precipitation hardening, fracture analysis, multistage solutionizing, artificial aging

## Abstract

The study aimed to compare and analyze the mechanical property and fracture behavior of LM4 composites reinforced with TiB_2_ (1–3 wt.%) and Si_3_N_4_ (1–3 wt.%) ceramic powders. A two-stage stir casting process was employed for the effective preparation of monolithic composites. To further enhance the mechanical properties of composites, a precipitation hardening treatment (both single (SSHT) and multistage (MSHT), followed by artificial aging at 100 and 200 °C) was conducted. From mechanical property tests, it was understood that in both the monolithic composites, the properties improved with an increase in wt.% of reinforcements, and composite samples subjected to MSHT + 100 °C aging treatment bested other treatments in terms of hardness and UTS values. Compared to as-cast LM4, there was a 32 and 150% increase in hardness and a 42 and 68% increase in UTS for as-cast and peak-aged (MSHT + 100 °C aging) LM4 + 3 wt.% TiB_2_ composites, respectively. Similarly, there was a 28 and 124% increase in hardness and a 34 and 54% increase in UTS for as-cast and peak-aged (MSHT + 100 °C aging) LM4 + 3 wt.% Si_3_N_4_ composites, respectively. Fracture analysis of the peak-aged composite samples confirmed the mixed mode of fracture in which brittle mode was dominating.

## 1. Introduction

LM4 is a cast Aluminum-Silicon (Al-Si) hypoeutectic alloy that is usually employed in the automotive industry for the preparation of cylinder heads, electrical fittings, and tooling, among other things. Major alloying elements in LM4 are Si and copper (Cu), which are responsible for their superior properties when matched to other cast Al alloys. The modification/spheroidization of eutectic Si and formation of Al_2_Cu (copper aluminide) phase during the precipitation hardening treatment are some of the microstructural changes that help in attaining better mechanical properties of the alloy [1]. External factors such as the addition of reinforcements/modifiers/grain refiners, the work hardening process, and heat treatment (HT) positively impact mechanical properties. Further modifications in LM4/Al-Si alloy can be accomplished by adding certain grain refiners and modifiers, which help in the transformation of Si (eutectic) from an acicular needle-like structure to a spherical form [2]. To enhance the hardness and to improve the creep lifetime of Al-Si alloy, Shan et al. [3] added multi-walled carbon nanotubes as reinforcements to Al-Si alloy, and from the results, they concluded that the addition of reinforcement caused improvement in hardness and creep lifetime. Mekala et al. [4] improved the properties of LM25 alloy by reinforcing it with high entropy alloy particulates, a stir casting method was used to fabricate the composites. From TEM analysis, they confirmed the absence of intermetallic phases at the interface and concluded that the reason for improving mechanical strength was purely because of the reinforcement. Similar modifications to the microstructures can be made by the inclusion of certain ceramic reinforcements. TiB_2_ and Si_3_N_4_ are of those ceramic reinforcements which can modify the microstructure of Al alloys. TiB_2_ ceramic reinforcement has a density of 4.52 g/cm^3^, a hardness of 2468 VHN, and a melting point of 2790 °C [5]. Similarly, Si_3_N_4_ has a density of 3.17 g/cm^3^, a hardness of 1800–2400 VHN, and a melting point of 1900 °C [6]. As the density of both these reinforcements is higher than that of the Al alloys, it is very difficult to fabricate composites with uniform reinforcement distribution. As per Pethuraj et al. [7], the best method to fabricate AMMCs was the stir casting method, which provided better bonding between the matrix and reinforcement material and also provided better distribution of reinforcements. Bernoulli et al. [8] used semisolid stirring and ultrasonic processing methods to overcome density-related issues by adding highly dense graphene powder to A319 alloy. From microstructural analysis, it was confirmed that using a semisolid stirring process, proper distribution of reinforcements can be achieved without any powder floating on the molten melt. Some literature related to the preparation of composites using TiB_2_ and Si_3_N_4_ as reinforcements and HTs performed to enhance the mechanical properties are discussed below.

Li et al. [9] in their study confirmed that adding TiB_2_ to Al-Si alloy caused α-Al grain refinement and modification of Si particles, which resulted in higher mechanical properties of the prepared composites. This grain refinement was mainly because of the heterogenous nucleation sites formed by the presence of TiB_2_ particles and the growth limitation of Ti which was dissolved from Al_3_Ti. Dipankar et al. [10] observed growth in dislocation density because of TiB_2_ particles in the Al2024 matrix, which caused enhancement in mechanical properties; also, Al grains were refined. The microstructural analysis confirmed that preheating TiB_2_ before adding it to molten melt helped in the uniform distribution of TiB_2_ in the matrix. Ramesh et al. [11] prepared AA7075 + TiB_2_ (4, 8, 12 wt.%) composites and compared the hardness of prepared composites with the alloy. Results revealed that the composite with 12 wt.% TiB_2_ exhibited the highest hardness when compared to AA7075 alloy; the presence of hard TiB_2_ particles and better interaction between reinforcement and matrix were some of the crucial aspects for enhancing hardness. Similar studies were conducted by many researchers on Al-TiB_2_ composites [5,12] and concluded that TiB_2_ could be used as an effective reinforcement for the fabrication of Al metal matrix composites (AMMCs).

Mohanavel et al. [13] fabricated AA7178 + Si_3_N_4_ composites using the stir casting method, and the results confirmed that the addition of Si_3_N_4_ caused grain alteration of Al, which was the key cause for its mechanical property enhancement. Adding Si_3_N_4_ reinforcements to the Al matrix resulted in an increment of mechanical properties of the fabricated composites; preheating of the Si_3_N_4_ improved its wettability with the Al matrix [14,15,16]. This inclusion of reinforcements improved the heat treatability of the base alloy by accelerating the aging kinetics and allowing secondary hardening due to intermetallic precipitation. Zhigang et al. [17] examined the aging behavior of Al6061 + Si_3_N_4_ composites. From the outcomes, the authors concluded that after solutionizing, modification in grain structure was not observed, whereas after aging ß″, intermetallic phase was observed, which resulted in improvement of mechanical properties. Additionally, an acceleration in aging kinetics was observed after addition of Si_3_N_4_ reinforcement. Xiu et al. [18] developed Al-Si_3_N_4_ composites with the pressure infiltration method, and the authors concluded that the properties of fabricated composites could be enhanced by subjecting them to T6 HT. As per Wang et al. [19], to dissolve Mg_2_Si and to modify eutectic Si into spherical form, Al-Si must be solutionized at 540 °C. Additionally, during aging, the inclusion of any foreign particles (reinforcements) acted as obstacles to the movement of dislocation during mechanical testing. It provided better mechanical properties to the treated composites. Akhil et al. [20] performed a precipitation hardening treatment on Al-Si alloy and concluded that when solutionized at 500 °C, spheroidization of eutectic Si was noticed, and artificial aging resulted in increments of mechanical properties of the alloy. The major goal of solution heat treatment (SHT) [21] and its importance in improving the mechanical properties were studied by Han et al. [1]. Precipitation hardening on Al alloys was performed by many researchers [22,23,24]. It was concluded that this process helped Al-Si alloy in attaining better mechanical properties.

Following an extensive assessment of the literature, it was understood that a substantial amount of research had been conducted on the use of TiB_2_ and Si_3_N_4_ as reinforcements for various Al alloys. The effects of reinforcement addition and the formation of intermetallic phases following HTs were also thoroughly investigated. Additionally, researchers have previously analyzed the influence of a solutionizing treatment and aging on the mechanical properties of composites; however, little study has been conducted on the influence of multistage solutionizing and aging. As a result, in our work, we examined the hardness and tensile strength of composite samples exposed to single and multistage solutionizing followed by aging. As well, the influence of TiB_2_ and Si_3_N_4_ on LM4 composite aging behavior was studied.

## 2. Methodology

**Figure 1:**
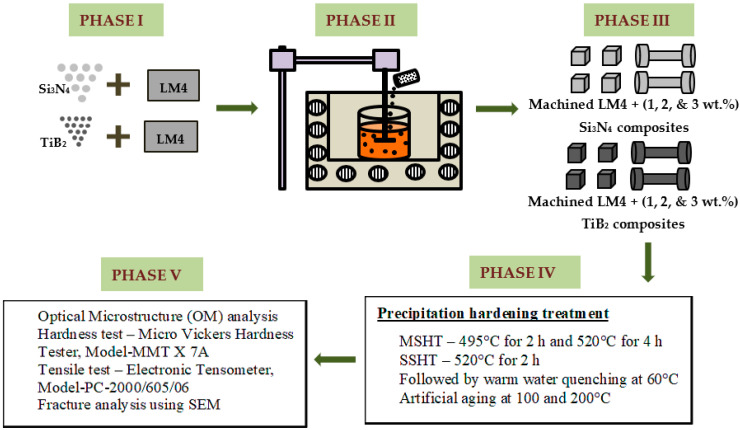
Methodology flow chart.

Figure 1 illustrates the detailed approach used in the current study, which is divided into five phases. Phase I—Materials and their properties, Phase II—Two-stage stir casting procedure, Phase III—Preparation of cast samples for further analysis, Phase IV—Precipitation hardening treatment, Phase V—Morphological analysis (using an optical microscope (OM) and a scanning electron microscope (SEM)) and mechanical property testing (hardness and tensile).

### 2.1. Phase I

For this investigation, LM4 was chosen as the matrix material, which was purchased from LM exchange, Coimbatore. The alloy composition of LM4 was determined as per ASTM E-1251-2011 [25] at BUREAU VERITAS (INDIA) PVT. LTD, Bangalore, and the major alloying elements include Si and Cu with 5.925 and 2.476 wt.%, and other alloying elements include Fe, Mn, Ni, Sn, and Mg with 0.6, 0.12, <0.03, 0.04, and 0.176 wt.%. As reinforcements, TiB_2_ (dark grey) and Si_3_N_4_ (light grey) were considered. Both reinforcements were bought from NANOCHEMAZONE INC, Canada. They have an average particle size of (TiB_2_—6.765 µm and Si_3_N_4_—26 µm), which was determined using a scanning electron microscope (SEM) [26,27]. Both TiB_2_ and Si_3_N_4_ are hard reinforcement particles with a hardness value of 2468 and 1750 VHN [28]; the unique properties of these hard reinforcement particles help in attaining enhanced properties of the cast composites when added separately to the LM4 matrix.

### 2.2. Phase II

A two-stage stir casting technique was used in this study for the preparation of composites. Initially, LM4 was melted in a furnace at 745 °C [29], which was the perfect melting temperature for Al-Si alloys. After the complete melting of LM4, a mechanical stirrer was submerged into the melt and stirred at 200 rpm/5 min. This initial stage of stirring was performed to reduce the melt temperature to 600 °C, to get the melt to a semisolid state. Meanwhile, both Si_3_N_4_ and TiB_2_ were preheated at 350 °C/30 min [27,30] and were added to the melt (while stirring) only after the melt temperature reached 600 °C (semisolid state). Mixing reinforcements at this semisolid state results in strong bonding with the matrix material [31], and preheating of reinforcements helps in removing the moisture content (if any) and helps in better uniform distribution within the molten LM4. Thereafter, the temperature was increased to 730 °C, and stirring was continued for an additional 10 min at 200 rpm to ensure proper distribution of reinforcements in the molten LM4. The composite molten mixture was then poured into preheated (500 °C/2 h) bar and pencil molds and left for solidification; molds were preheated to avoid rapid solidification of the melt [32]. The following composites were prepared, and short forms of the cast composites are mentioned in Table 1.

### 2.3. Phase III

Cast composites collected from bar molds were machined into small cubes for microstructural analysis, HT, and hardness testing using wire EDM. Pencil mold cast composites were used to prepare tensile specimens as per ASTM-E8M standards [33] using a CNC turning machine. Before microstructure analysis and hardness testing, the cube samples were polished using a METKON-FORCIPOL-02 polishing machine.

### 2.4. Phase IV

Cast composite samples were subjected to a precipitation hardening treatment. This was carried out in two stages. In stage 1, one set of samples from both the composites was subjected to MSHT, followed by water quenching at 60 °C to reduce the internal stresses. Then, the samples were treated for artificial aging at 100 and 200 °C. Samples were taken out every 30 min, quenched in water at room temperature, and a hardness test was carried out until peak hardness was achieved. Similarly, in stage 2, separate samples from both composites were subjected to SSHT, followed by quenching and artificial aging at 100 and 200 °C until peak hardness was achieved.

### 2.5. Phase V

Optical microstructure (OM) analysis was done using OLYMPUS U-TV0.5XC-3 (BX53M). Using this reinforcement distribution and different phase formations, modifications that occurred in the heat-treated composites were identified. The hardness test was performed using a Micro Vickers Hardness tester, Model-MMTX7A, as per ASTM E384 standards [34]. During this test, the load was set at 200 gmf with a dwell time of 15 s. Five readings were taken for each sample, and the average hardness value was used in the graphs plotted along with the standard deviation. Hardness tests were carried out for as-cast (AC) samples and samples subjected to SSHT and MSHT, followed by artificial aging at 100 and 200 °C for different time intervals (0–16 h) until peak hardness was attained. A tensile test was performed only on AC and peak-aged (highest VHN value) samples of both the composites using an Electronic Tensometer, Model-PC-2000/605/06, as per ASTM E8 standards [33]. The load was maintained at 20 kN with a break test mode and speed of 1 mm/min. Fracture analysis for the broken tensile samples of both AC and peak-aged (highest VHN value) samples of both composites was performed using SEM, and the mode of fracture experienced by them was illustrated.

## 3. Results and Discussion

### 3.1. Optical Microstructure Analysis

**Figure 2:**
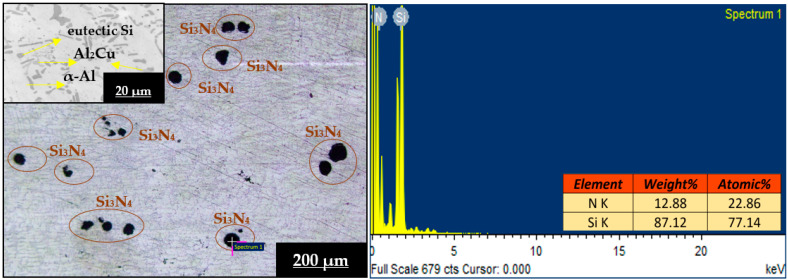
OM images and EDS analysis of L3SN heat-treated sample.

**Figure 3:**
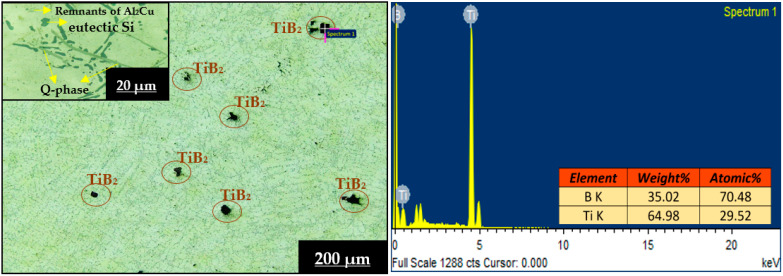
OM images and EDS analysis of L3TB heat-treated sample.

Figure 2 and Figure 3 depict OM images (with different magnifications) and EDS analysis of L3SN and L3TB heat-treated (peak-aged) composite samples. From Figure 2, we can see that the reinforcement distribution is homogeneous throughout the matrix, and proper bonding can be observed with almost zero porosity. Here, dark spots are identified as Si_3_N_4_ reinforcement particles, and the EDS report also confirms the presence of Si and N peaks of spectrum 1 (spot analysis). At some places, small dark spots are observed, identified as small reinforcement powder fragments. This proper distribution of reinforcement indicates that the two-stage stir casting method can be effectively used in the preparation of the composites. The magnified image of L3SN in Figure 2 shows the presence of eutectic Si (circular shape), which indicates that the addition of Si_3_N_4_ as reinforcement caused a modification in eutectic Si; also the formation of Al_2_Cu (copper aluminide) light and blocky phase is observed [35], which is the main reason in attaining peak hardness after precipitation hardening treatment. Similarly, from Figure 3, we can see that the distribution of TiB_2_ within the matrix is uniform, and no porosity is observed; also, the EDS report confirms the peaks of Ti and B of spectrum 1 (spot analysis). From the magnified image of L3TB in Figure 3, we can observe the better modification of eutectic Si when compared to L3SN; also, the addition of TiB_2_ causes more Al grain refinement, and more nucleation sites are formed during HT process. Along with Al_2_Cu, the Q-Al_5_Mg_8_Cu_2_Si_6_ phase also precipitated here, with a long needle shape rather than the uneven form seen in the LM4 microstructures; also, some remnants of Al_2_Cu are observed [23]. The presence of these two phases helped TiB_2_ composites achieve better mechanical properties compared to Si_3_N_4_ composites. From the microstructures, it can be confirmed that there is no slag nor porosity, which indicates the soundness of the two-stage stir casting method. Formation of metastable intermetallic phases (Al_2_Cu, Q, and Mg_2_Si) is validated by XRD analysis from prior research [26,27,36], which are also in line with the literature.

### 3.2. Micro Vickers Hardness Comparison

**Figure 4:**
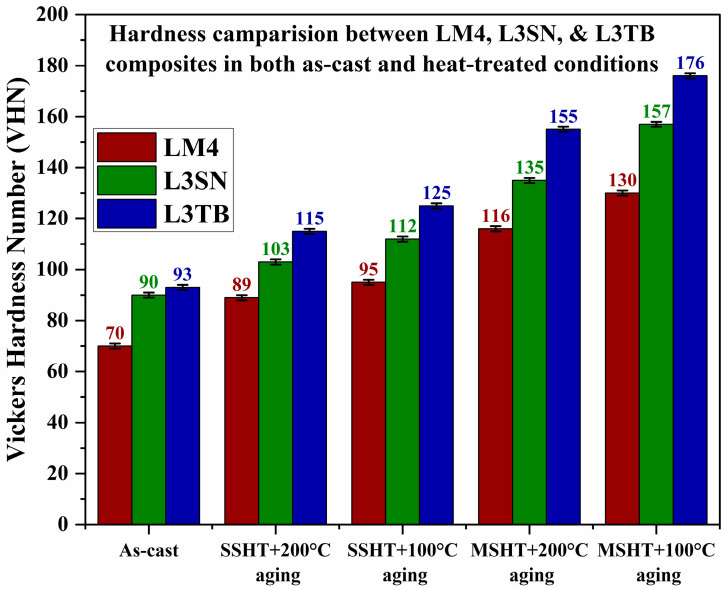
Hardness comparison between LM4, L3SN, and L3TB in AC and heat-treated conditions.

**Figure 5:**
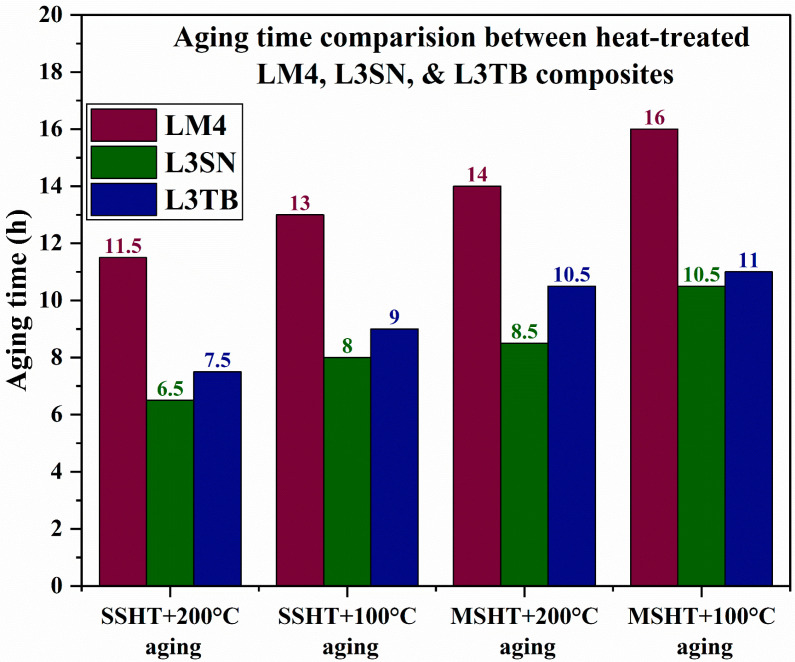
Peak aging time comparison between LM4 and its composites.

A hardness comparison of LM4 and its composites in both AC and heat-treated conditions is shown in Table 2. Hardness values of cast composites in AC conditions are higher than that of alloy because of the presence of reinforcements in the matrix [15], and TiB_2_ composites displayed highest hardness values in AC and heat-treated conditions compared to Si_3_N_4_ composites; the presence of TiB_2_ reinforcement particles caused refinement in Al grains and helped in the creation of more dislocations, which resulted in better hardness of its composites. Jian et al. [37] performed a similar study wherein they used TiB_2_ as reinforcement for Mg-Al-Si alloy. The TiB_2_ addition caused grain refinement and helped in the swift formation of intermetallic phases, which caused the improvement of hardness and tensile strength values of the prepared composites. The hardness of composites increased linearly with an increase in wt.% of reinforcements in both cases. From Table 2, we can observe that the heat-treated alloy and composites achieved better hardness than AC alloy and composites. After HT, because of the formation of metastable phases like Al_2_Cu, Q, and Mg_2_Si [36], the samples exhibited better hardness than AC samples. Similar observations were made by Bharat et al. [38] wherein precipitation-hardened samples of Al 2014 composite showed almost 32% improvement in hardness when compared to AC samples. Samples subjected to MSHT achieved the highest hardness than samples subjected to SSHT because of the presence of more precipitates [26]. Figure 4 depicts the VHN values of LM4, L3TB, and L3SN in both AC and heat-treated conditions. At every stage, L3TB samples dominated the hardness values compared to LM4 and L3SN. Figure 5 depicts the time taken by the alloy and its composites to attain peak hardness. Samples aged at 100 °C achieved greater hardness (Figure 4) values than samples aged at 200 °C, but the time taken to reach peak hardness is more in the case of 100 °C aging as shown in Figure 5. This phenomenon can be explained by the help of aging kinetics [39]. We can observe a reduction in aging time in composites when compared to alloy, and this is because the presence of reinforcement particles accelerates the aging kinetics. With an increase in wt.% of TiB_2_ and Si_3_N_4_, the aging time needed to attain peak hardness is reduced. Compared to TiB_2_, the composites time taken to reach peak hardness by Si_3_N_4_ composites is less, as shown in Figure 5, but the peak hardness value of TiB_2_ composites is more when compared to Si_3_N_4_ composites, as shown in Figure 4.

### 3.3. Tensile Strength Analysis

**Figure 6:**
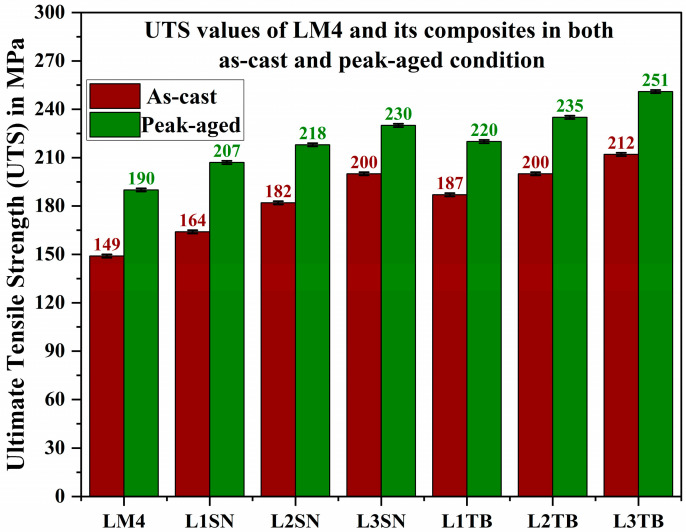
UTS values of AC and peak-aged LM4 and its composites.

Figure 6 shows UTS values of LM4 and its composites in AC and peak-aged conditions. Composites displayed better UTS values than LM4. The addition of reinforcements caused the formation of dislocation pile-up, and the presence of these hard reinforcement particles obstructed dislocation motion and absorbed most of the stress during testing, with an increase in wt.% UTS values improved in both the composites. TiB_2_ composites have the highest UTS compared with Si_3_N_4_ composites, the explanation of which is similar to that of hardness values. Table 3 shows the load vs. displacement values of AC and peak-aged LM4, L3TB, and L3SN samples. We can notice that peak-aged L3TB could bare more load till 8110 N before breakage than any other sample. These values justify the UTS values shown in Figure 6.

### 3.4. Fracture Analysis of Peak-Aged L3TB and L3SN

**Figure 7:**
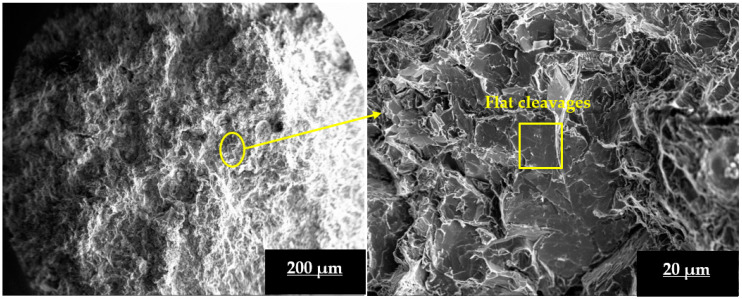
Fracture surface SEM image of peak-aged L3TB composite sample at different magnifications.

**Figure 8:**
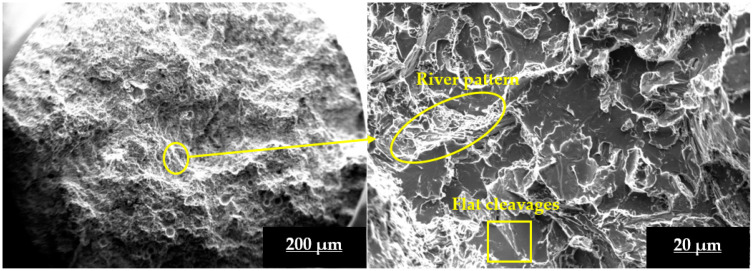
Fracture surface SEM image of peak-aged L3SN composite sample at different magnifications.

Fracture analysis of LM4 in both AC and peak-aged conditions was performed in our earlier studies [26]; from SEM images, it was understood that in AC condition, the LM4 sample experienced ductile failure, whereas in peak-aged condition (MSHT + 100 °C aging), samples experienced a mixed mode of failure (both ductile and brittle) because it had displayed a better UTS value than AC LM4. The fracture analysis of peak-aged L3TB and L3SN is performed in this study, and the SEM images of the same are given in Figure 7 and Figure 8, respectively. The existence of flat cleavages can be observed from the magnified SEM image of Figure 7, indicating brittle fracture. Furthermore, fine dimples are observed, which might have transitioned from coarse to fine during HT, indicating brittle fracture. In the instance of LM4, the dimple density was higher, but there were also micro-voids and some river patterns, indicating that the sample had undergone ductile failure. One of the key causes for the brittle mode of fracture and the highest UTS value in peak-aged L3TB sample is due to the existence of hard reinforcements and the formation of metastable intermetallic phases during HT [40].

Figure 8 depicts a fracture surface analysis of a peak-aged L3SN sample at various magnifications. The combination of flat cleavages and river patterns in the magnified image of a peak-aged L3SN sample indicates a mixed mode of fracture, with brittle mode dominating. Si_3_N_4_ could not refine the grain size of α-Al and could not modify eutectic Si to the same extent as TiB_2_ could. However, because of the existence of hard ceramic reinforcements and intermetallic phases, peak-aged L3SN samples experienced mixed mode of failure with greater UTS values than that of peak-aged LM4.

## 4. Conclusions

- Two-stage stir casting method was proven to be efficient in fabricating defect-free composites with uniform distribution of reinforcements within the matrix.
- With an increase in wt.% of reinforcements, there was an enhancement in the mechanical properties of composites in both cases (TiB_2_ and Si_3_N_4_).
- TiB_2_ composites achieved better hardness and UTS values when compared to Si_3_N_4_ composites in both AC and heat-treated conditions.
- Precipitation hardening treatment was effective enough to improve the properties of composite samples when compared to AC samples.
- Samples exposed to MSHT + artificial aging at 100 °C gave the highest hardness and UTS values compared to MSHT + artificial aging at 200 °C, and SSHT + artificial aging at 100 and 200 °C samples in both the composites.
- The formation of metastable intermetallic phases (Al_2_Cu, Q, and Mg_2_Si) was the key factor for the enhancement of mechanical properties of heat-treated composites.
- Fracture surface analysis revealed a brittle failure in the peak-aged L3TB sample, whereas the peak-aged L3SN sample experienced a mixed mode of failure.

## Figures and Tables

**Table 1:** Abbreviation list.

Composites Prepared	Short Name
LM4 + 1 wt.% TiB_2_	L1TB
LM4 + 1 wt.% Si_3_N_4_	L1SN
LM4 + 2 wt.% TiB_2_	L2TB
LM4 + 2 wt.% Si_3_N_4_	L2SN
LM4 + 3 wt.% TiB_2_	L3TB
LM4 + 3 wt.% Si_3_N_4_	L3SN

**Table 2:** Vickers hardness number (VHN) comparison between LM4 and its composites in AC and peak-aged condition along with peak aging time in hours (h).

Material	AC	SSHT + 100 °C Peak-Aged	SSHT + 200 °C Peak-Aged	MSHT + 100 °C Peak-Aged	MSHT + 200 °C Peak-Aged
LM4	70	95 (13 h)	89 (11.5 h)	130 (16 h)	116 (14 h)
L1SN	87	99 (10 h)	94 (8 h)	135 (12 h)	120 (9.5 h)
L2SN	88	105 (9 h)	100 (7.5 h)	143 (11.5 h)	125 (9 h)
L3SN	90	112 (8 h)	103 (6.5 h)	157 (10.5 h)	135 (8.5 h)
L1TB	89	108 (11 h)	100 (9.5 h)	145 (14 h)	126 (12 h)
L2TB	91	117 (10 h)	107 (8.5 h)	160 (12.5 h)	140 (11.5 h)
L3TB	93	125 (9 h)	115 (7.5 h)	176 (11 h)	155 (10.5 h)

**Table 3:** Load vs. Displacement values of LM4, L3SN, and L3TB composites in both AC and peak-aged condition.

Material	AC Condition		Peak-Aged Condition	
	Displacement (mm)	Load (N)	Displacement (mm)	Load (N)
LM4	1.25	4834	1.935	6011
L3SN	1.9	6482	2.0	7453
L3TB	1.975	6992	2.22	8110

## Data Availability

Not applicable.
